# Direct Activation of Human Dendritic Cells by Particle-Bound but Not Soluble MHC Class II Ligand

**DOI:** 10.1371/journal.pone.0063039

**Published:** 2013-05-02

**Authors:** Renato B. Baleeiro, Karl-Heinz Wiesmüller, Lars Dähne, Jürgen Lademann, José A. Barbuto, Peter Walden

**Affiliations:** 1 Charité – Universitätsmedizin Berlin, Humboldt-Universität zu Berlin and Freie Universität Berlin, Department of Dermatology, Venerology and Allergology, Berlin, Germany; 2 Department of Immunology, Institute of Biomedical Sciences, University of São Paulo, São Paulo, Brazil; 3 Eberhard-Karls-Universität Tübingen, Tübingen, Germany; 4 EMC microcollections GmbH, Tübingen, Germany; 5 Surflay Nanotec GmbH, Berlin, Germany; New York University, United States of America

## Abstract

Dendritic cells (DCs) are key activators of cellular immune responses through their capacity to induce naïve T cells and sustained effector T cell responses. This capacity is a function of their superior efficiency of antigen presentation via MHC class I and class II molecules, and the expression of co-stimulatory cell surface molecules and cytokines. Maturation of DCs is induced by microbial factors via pattern recognition receptors such as Toll-like receptors, pro-inflammatory cytokines or cognate interaction with CD4^+^ T cells. Here we show that, unexpectedly, the PanDR helper T cell epitope PADRE, a generic T helper cell antigen presented by a large fraction of HLA-DR alleles, when delivered in particle-bound form induced maturation of human DCs. The DCs that received the particle-bound PADRE displayed all features of fully mature DCs, such as high expression of the co-stimulatory molecules CD80, CD86, CD83, the MHC-II molecule HLA-DR, secretion of high levels of the biologically active IL-12 (IL-12p70) and induction of vigorous proliferation of naïve CD4^+^ T cells. Furthermore, the maturation of DCs induced by particle-bound PADRE was shown to involve sphingosine kinase, calcium signaling from internal sources and downstream signaling through the MAP kinase and the p72syk pathways, and finally activation of the transcription factor NF-κB. Based on our findings, we propose that particle-bound PADRE may be used as a DC activator in DC-based vaccines.

## Introduction

Dendritic cells (DCs) are the most efficient and most important antigen presenting cells (APCs) for T cell priming and induction of adaptive immune responses [1]. They reside in peripheral tissues in an immature state and sample their microenvironment by taking up and processing antigens [2]. Exposure of DCs to inflammatory mediators such as TNF-α, IL-1-β and IL-6 or microbial agents such as TLR agonists induces their maturation with phenotypical and functional changes. The DC activation marker CD83 [3], [4] and receptors for chemokines from secondary lymphoid organs such as CCR7 [5] are expressed and the co-stimulatory molecules CD40, CD80 and CD86 are up-regulated [6]. In addition, fully mature DCs produce pro-inflammatory cytokines, in particular interleukin (IL)-12, which plays critical roles in the induction of efficient CD4^+^ T helper cell (Th) and sustained effector CD8^+^ T cell immune responses characterized by high interferon (IFN)-γ production and cytotoxicity [7]. These changes enable DCs to induce effective adaptive immune responses. Because of their unique capacities as antigen-presenting cells, DCs are widely used in cancer immunotherapy and loaded with tumor-associated antigens as cellular vaccines [8], [9]. The antigens administered hereby are often synthetic peptides such as epitopes for CD8^+^ effector T cells. To render such peptides immunogenic and enhance the potency of the vaccines, CD4^+^ T cell help is believed to be important in the initiation of CD8^+^ effector T cell responses and for establishing T cell memory [10]. In addition to specific MHC class II epitopes, recall antigens and Pan HLA-DR epitopes (PADREs) have been explored as helper T cell antigens. PADREs are designed to bind to many different HLA-DR and thereby to help overcoming the problem of identifying helper antigens that match the HLA class II of a particular patient [11]. PADREs have been used together with different vaccine antigens for induction of cytotoxic effector T cells in preclinical models [11]–[15] as well as in clinical trials [15], [16]. To ensure effective induction of DC maturation, combinations of antigens for coordinated induction of helper and effector T cell responses are often given together with TLR agonists or pro-inflammatory cytokines as drivers of functional differentiation of DC and induction of co-stimulatory cytokines and cell surface receptors. Moreover, the design of vaccines for effective induction of T cell-mediated immunity requires, in addition to the before-mentioned, carrier systems for transport of the essential vaccine constituents to and their timely release at dendritic cells. To this avail we investigated the effects of different essential vaccine constituents on the induction of prophylactic and therapeutic T cell-based immune responses. Unexpectedly, particle-bound in contrast to soluble PADRE was discovered to induce phenotypic and functional differentiation of immature into fully mature DCs with strong T cell-stimulatory capacity.

## Materials and Methods

### Peptides, Lipopeptides and Peptide-loaded Particles

The peptides aK-(L-Cha)-VAAWTLKAAa-Aca-C (PADRE), influenza matrix protein M1_58–66_ GILGFVFTL (GILG) and CMV pp65_595–603_ NLVPMVATV (NLVP) as well as di-acyl-lipopeptide Pam_2_Cys-GDPKHPKSF (P2C) were synthesized with C-terminal tetralysine tags and attached to layer-by-layer coated silica beads [17] of 1 µm diameter via negative charges on the outermost polymer layer.

### DC Generation and Stimulation

DCs were produced from PBMC using a protocol modified from [18]. In brief, PBMC from healthy donors were isolated from buffy coats by Ficoll gradient centrifugation. Cells of the interface were collected and plated in 6 well plates. Monocytes were allowed to adhere. After 2 h, non-adherent cells were washed off and the adherent cells were cultured for 7 days, in serum free AIM-V culture medium (Invitrogen, Carlsbad, CA, USA) with GM-CSF (50 ng/mL, R&D Systems, Minneapolis, MN, USA) and IL-4 (50 ng/mL, R&D Systems, Minneapolis, MN, USA). In some experiments, prior to adherence, the PBMCs were depleted of T cells by magnetodepletion of CD3+ cells (Miltenyi Biotec GmbH, Bergisch Gladbach, Germany), following the manufacturer’s instruction. The cultures were kept in a CO_2_ incubator in a humidized atmosphere with 8% CO_2_ at 37°C. On day 5 of culture, empty particles, particles loaded with peptides as indicated or free peptides, LPS (1 µg/mL; Sigma-Aldrich, Germany), ionomycin (1 µg/mL; Sigma-Aldrich, Germany) or an inflammatory cytokine cocktail of IL-1β (10 ng/mL), IL-6 (25 ng/mL) TNF-α (10 ng/mL; all Strathmann Biotech, Hamburg, Germany) were added and the culture continued. Where inhibitors were used, cells were incubated for 2 hours at 37°C with the respective inhibitor prior to the addition of the maturation stimulus. The specific inhibitors used were SB203580 for p38 MAPK at 20 µM, triptolide for NF-κB at 100 nM, LY294002 for Pl3K at 10 µM (all Invivogen, San Diego, Ca, USA), Piceatannol for p72Syk at 25 µM and CK59 for CaMKII at 50 µM (all Calbiochem, Darmstadt, Germany), BAPTA-AM (membrane-permeating calcium chelator) at 10 µM, D609 for PLC at 100 µM, SKI-II for SK at 10 µM (all Sigma-Aldrich, Germany) and cyclosporine A for calcineurin (Fluka Chemie GmbH, Steinheim, Germany) at 2 µM. Cells were harvested on day 7 and analyzed by flow cytometry or used for co-cultures with T cell.

### CD4^+^ T Lymphocytes Assays

Naïve and total CD4^+^ T lymphocytes were isolated from peripheral blood by magnetosorting. First, CD4^+^ T cells were isolated from the PBMCs by negative selection using a commercial kit (Miltenyi Biotec GmbH, Bergisch Gladbach, Germany) following the manufacturer’s instruction. In some experiments the memory/effector population was removed by negative selection of the CD45RO^+^ population with PE-labeled monoclonal antibody against the CD45RO (BD Bioscience, Chicago) and magnetic beads with antibodies to PE (Miltenyi Biotec GmbH, Bergisch Gladbach, Germany). The untouched CD45RA^+^ naïve T lymphocytes were labeled with 10 µM CFSE [19] in PBS with 0.1% BSA for 10 min at 37°C followed by 5 min at 4°C. Then the cells were washed twice in PBS and cultured together with the autologous DCs in 96 wells plates of 1×10^4^ DCs with 2×10^5^ T lymphocytes for 7 days, then harvested and analyzed by flow cytometry.

### Flow Cytometry

To determine DC states and activation, the cell suspensions were labeled with fluorescent monoclonal antibodies against Lin (cocktail of FITC-conjugated antibodies against the molecules CD3, CD14, CD19 and CD56) for exclusion of non-DCs, CD11c, CD40, CD80, CD83, CD86 and HLA-DR (Caltag Laboratories, Burlingame, California or BD Bioscience, Chicago). T lymphocytes were labeled with antibodies for CD4, CD45RA and CD45RO. Proliferation of the CD4^+^ T cells was determined by flow cytometry after labeling of the cells with CFSE and co-culture with the differently treated DC. All stained cells were analyzed with a FACSCalibur flow cytometer (Becton Dickinson, San Jose, California). The data were processed and displayed using the CellQuest software (Becton Dickinson, San Jose, California) or WinMDi (Purdue University, USA; www.purdue.edu).

### Measurement of Intracellular Ca^2+^

Intracellular Ca^2+^ was measured as described [20] with some modifications. Briefly, DCs were loaded with 5 µM Fluo-3/AM (Calbiochem, Darmstadt, Germany) by incubation at 37°C for 30 min under shaking and protection from light. The Fluo-3/AM-loaded DCs were centrifuged and washed two times. Then the cells were suspended in culture medium with or without EDTA (10 mM) and incubated protected from light with empty particles, particle-bound PADRE, particle-bound P2C, soluble LPS (1 µg/mL) or ionomycin (1 µg/mL) for 10 min at 37°C and analyzed by flow cytometry for Ca^2+^-dependent fluorescence.

### ELISA for IL-12p70

The supernatants of the DC cultures were collected after 48 h incubation with the indicated stimuli and stored at –20°C. IL-12p70 levels were measured by ELISA Max Standard kit (BioLegend) in 96-well microtiter plates according to the manufacturer’s instructions. Results are expressed as pg/mL.

### Statistical Analysis

Results were tested for normality by Kolmogorov–Smirnov test. Comparisons of the results obtained from the different experimental groups were done by ANOVA. Differences with a p<0.05 were considered significant. All statistical analyses were performed using the Graphpad Software Prism 2.01 for Windows.

### Ethics Statement

The reported study was reviewed and approved by the institutional ethics committee of the Charité – Universitätsmedizin Berlin (Protocol # EA1/148/08) and the human materials used with written informed consent.

## Results

### Particle-bound but not Soluble PADRE Induces DC Maturation

**Figure 1:**
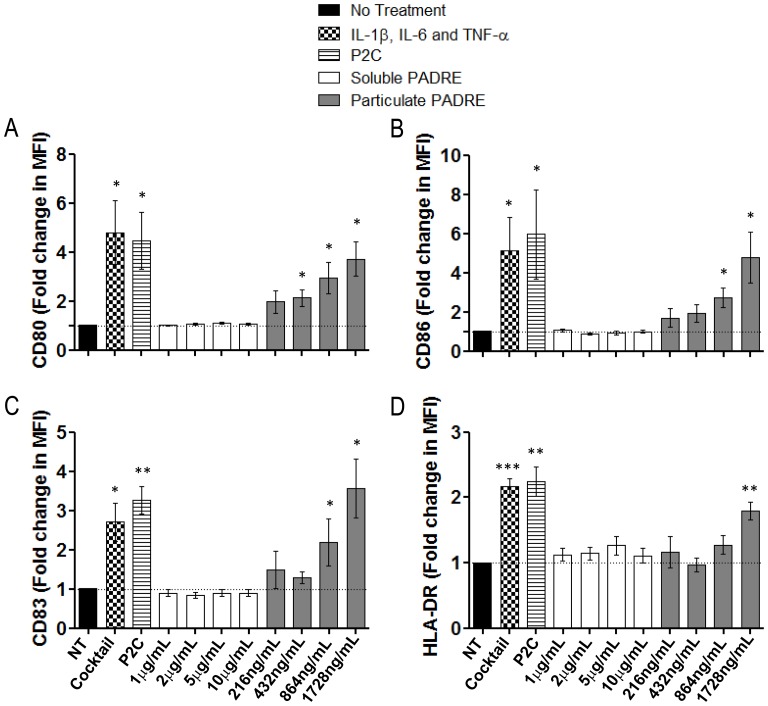
Phenotypic maturation of dendritic cells (DCs) induced by particles coated with PADRE. iDCs were generated from adherent PBMCs depleted of T cells. The adherent cells were cultured in serum-free AIM-V medium with GM-CSF and IL-4, harvested on day 5 and incubated with soluble PADRE, particle-bound PADRE or an inflammatory cytokine cocktail of IL-1β, IL-6 and TNF-α for 2 additional days. On day 7 the cells were labelled with antibodies for the HLA-DR, CD11c and Lin (cocktail of FITC-conjugated antibodies against the molecules CD3, CD14, CD19 and CD56) for exclusion, and CD80, CD83 and CD86, and analyzed by flow cytometry. The graphs show mean fold changes with standard error of the expression of CD80 (**A**), CD86 (**B**), CD83 (**C**), and HLA-DR (**D**). n = 5; NT: no treatment; Cocktail: IL-1β, IL-6 and TNF-α. * = p<0.05; ** = p<0.01; and *** = p<0.001 comparing the group treated with the indicated compound vs. the group “NT”.

**Figure 2:**
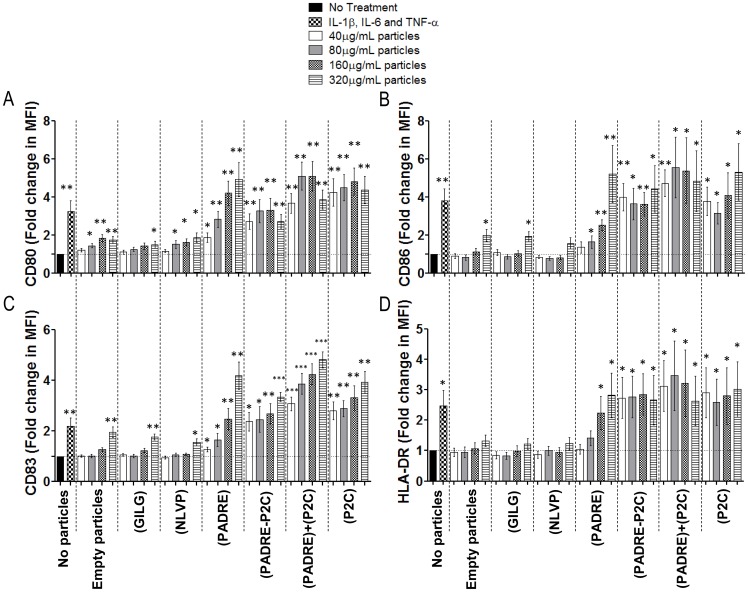
Specificity of the DC-inducing activity of particle-bound PADRE. iDCs as in the experiments shown in Figure 1 were incubated either without stimuli (negative control) or with an inflammatory cytokine cocktail of IL-1β, IL-6 and TNF-α as positive controls, or with particles without cargo or loaded with GILGFVFTL peptide, NLVPMVATV peptide, PADRE, diacyl-lipopeptide P2C, a mix of both PADRE and P2C or with a mixture of particles carrying either PADRE or P2C. After two days cultures the cells were labelled with antibodies for the CD11c and Lin (cocktail of FITC-conjugated antibodies against the molecules CD3, CD14, CD19 and CD56) for exclusion, and CD80, CD83, CD86 and HLA-DR, and analyzed by flow cytometry. The graphs show fold of expression of the molecules CD80 (**A**), CD86 (**B**), CD83 (**C**) and HLA-DR (**D**) with standard error. n = 6. * = p<0.05; ** = p<0.01; and *** = p<0.001 comparing the group treated with the indicated compound vs. the group “No Treatment”.

In studies aimed at the development of delivery systems that target vaccine antigens efficiently to dendritic cells in an immunogenic fashion, we have been testing particles surface-coated with different compounds. Among these compounds, we have tested CD8 and CD4 T cell epitopes (viral epitopes for CD8 and Pan-DR for CD4 T cells) and a TLR2/6-agonist, the dipalmitoyl lipopeptide (P2C). Our experimental set-up relies on the assessment of the effect of such compounds on immature dendritic cells (iDCs) generated from plastic-adherent human peripheral blood monocytes by incubation in medium with IL-4 and GM-CSF. Unexpectedly, particles with PADRE alone were found to efficiently induce maturation of iDCs. Such an effect was first evidenced by the expression of the DC activation marker CD83 and up-regulation of the co-stimulatory molecules CD80 and CD86, and MHC class II (Figure 1). This effect was dependent on PADRE bound to particles, as the free soluble peptide was inactive. Maturation of iDCs was not caused by the particles themselves, as neither particles without peptides nor particles coated with other peptides such as the influenza matrix protein epitope GILGFVFTL or the CMV pp65 epitope NLVPMVATV had any effect (Figure 2). The latter two peptides are MCH class I-binders, specifically HLA-A*0201-restricted T cell epitopes. No activation of DCs by particles bearing these peptides was seen in experiments with iDCs prepared from 20 different HLA-A*0201-positive donors (data not shown). The DC-stimulating effect of particle-bound PADRE thus seems to be mediated through MHC class II, not MHC class I. The magnitude of the DC response was comparable to the effects of the potent immunostimulatory TLR agonist P2C or a cocktail of the pro-inflammatory cytokines IL-1β IL-6 and TNF-α (Figure 1 and 2). PADRE appears to trigger a maximal cellular response of the DCs, as the addition of P2C either together with PADRE on the same particles or on separate particles did not further enhance the expression of activation and co-stimulatory markers by the DCs. Rather, it seems that the combination of PADRE and the TLR agonist reduces the effect of either one of these factors. Figures 1 and 2 summarize experiments done with cells from 5 and 6 independent donors, respectively, that all produced comparable results.

To exclude that T cells contaminating the iDCs preparations would cause activation of the DCs through cognate interaction with CD4^+^ T cells, we eliminated T cells from the DCs cultures prior to addition of the particles. As shown in Figure S1A and B, the elimination of T cells had no effect on the DC-stimulatory capacity of PADRE-loaded particles. Also the DC activation marker and co-stimulatory molecule CD40, which normally is induced upon cognate interaction of iDCs with CD4^+^ T cells and which via CD40-CD40L interaction leads to mutual activation of the interacting cells [21], was induced to comparable levels disregard of whether or not T cell had been eliminated prior to addition of the particles (Figure S1 B). These results were reproduced with cells from 4 independent donors in independent experiments.

### DCs Matured through Interaction with Particle-bound PADRE Stimulate Naïve T cells

**Figure 3:**
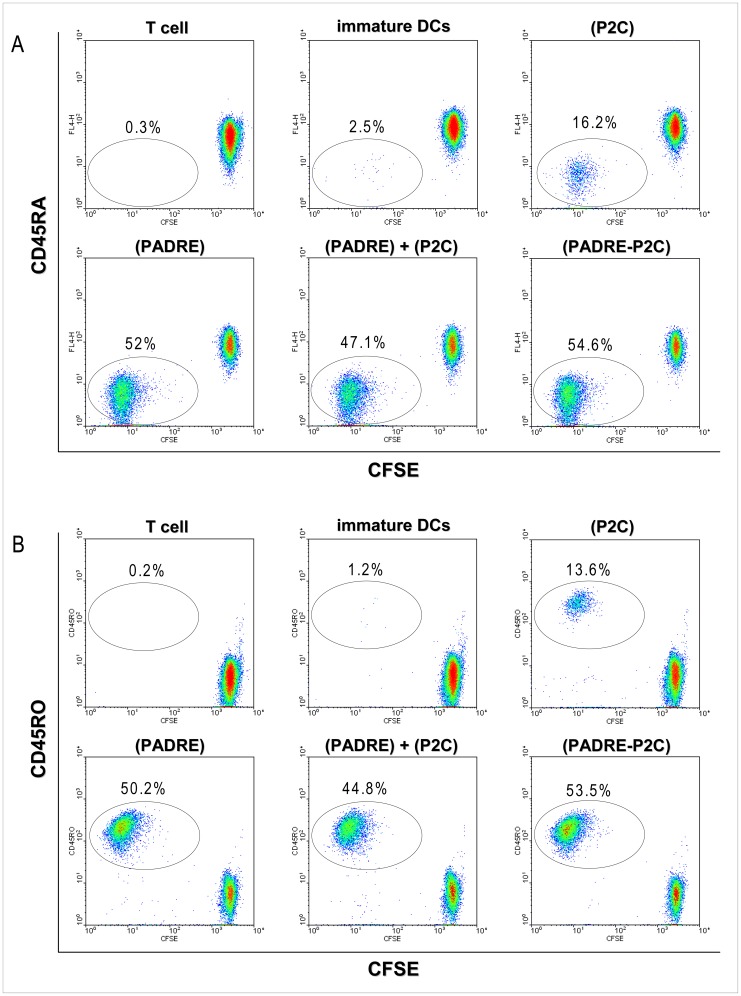
DCs stimulated with particle-bound PADRE induce vigorous proliferation of naïve CD4^+^ T cells. Adherent PBMCs previously depleted of T cells were cultured in presence of GM-CSF and IL-4 for five days and matured by incubation with particles carrying PADRE, P2C, a mixture of both, or a mixture of particles carrying either PADRE or P2C. After two days, the DCs were washed characterized by flow cytometry as for the experiments shown in Figures 1 and 2 and then co-cultured with CFSE-labelled naïve CD4^+^ T cells. After seven days of co-culture, the cells were harvested and labelled with antibodies to CD4, CD45RA and CD45RO and analyzed by flow cytometry. Proliferation of the T cells was determined by the reduction of CFSE upon successive cell divisions. The plots show CFSE and CD45RA (**A**) or CD45RO (**B**) on gated CD4^+^ T cells of one of five independent experiments with cells from five different healthy donors.

DCs stimulated with PADRE-loaded particles appeared to be functionally fully mature. Incubation of naïve T cells (CD3^+^CD4^+^CD45RA^+^CD45RO^−^) with DCs maturated with particle-bound PADRE induced not only differentiation of the naïve T cells into effector/memory T cells (CD3^+^CD4^+^CD45RA^−^CD45RO^+^) but also vigorous proliferation of these cells, as determined by flow cytometry by the dilution of the fluorescent dye CFSE upon successive cell divisions (Figure 3). These results were reproduced in 5 independent experiments with cells from different donors and are summarized in Figure S2. The experiments were done with iDCs rigorously depleted of T cells before particles were added.

### Maturation of DCs Induced by Particle-bound PADRE Involves Intracellular Calcium Signaling

**Figure 4:**
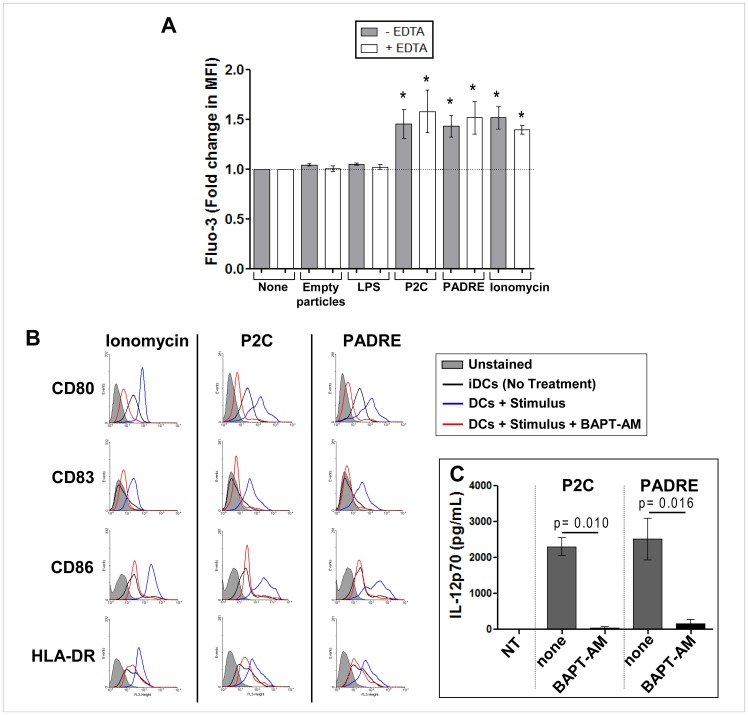
Particle-bound PADRE triggers DC maturation by inducing release of calcium from intracellular sources. Mobilization of intracellular calcium in DCs induced by particles carrying PADRE. iDCs generated as in the experiments shown in Figure 1 and 2 were harvested on day 5 and labelled with Fluo-3/AM. The DCs were then suspended in medium with or without EDTA and incubated for 10 minutes with empty particles, particle-bound PADRE, particle-bound P2C, soluble LPS or ionomycin and analyzed by flow cytometry. The graph shows mean fold changes of Fluo-3 fluorescence with standard error of independent experiments done with cells from three to five different healthy donors (**A**)**.** *indicates p<0.05– none vs. stimulus. In (**B–C**) is shown the effect of the blockade of intracellular calcium on the phenotypic maturation and IL-12p70 secretion of DCs induced by particle-bound PADRE. 5-day DCs were harvested, labelled or not with BAPTA-AM and incubated with particle-bound PADRE, particle-bound P2C or ionomycin for 2 more days. On day 7 the cells were labelled with antibodies for the CD80, CD86, CD83 and HLA-DR, and analyzed by flow cytometry. A representative experiment is shown (**B**). The supernatant of the stimulation cultures were collected and analyzed for IL-12p70 by ELISA (**C**). n = 3.

To elucidate the cellular mechanisms involved in the induction of DC maturation by particle-bound PADRE, we analyzed the mobilization of intracellular calcium using Fluo-3 as fluorescent calcium indicator. Particle-bound PADRE induced an increase of intracellular calcium, which was not seen in the DCs exposed to empty particles (Figure 4A). The calcium appears to come from intracellular stores as depletion of extracellular calcium in the culture medium with EDTA neither did affect the intracellular increase nor the activation of the DC (Figure 4A). Blockade of intracellular calcium using the BAPT-AM, a membrane-permeable selective chelator of intracellular Ca^2+^, completely abrogated the maturation of the DCs (Figure 4B and C), as evident from the unaltered low levels of the co-stimulatory molecules CD80 and CD86, the DC activation marker CD83, MHC class II HLA-DR and complete absence of IL-12p70 secretion.

### Sphingosine Kinase but not PLC Mediates Maturation of DCs

**Figure 5:**
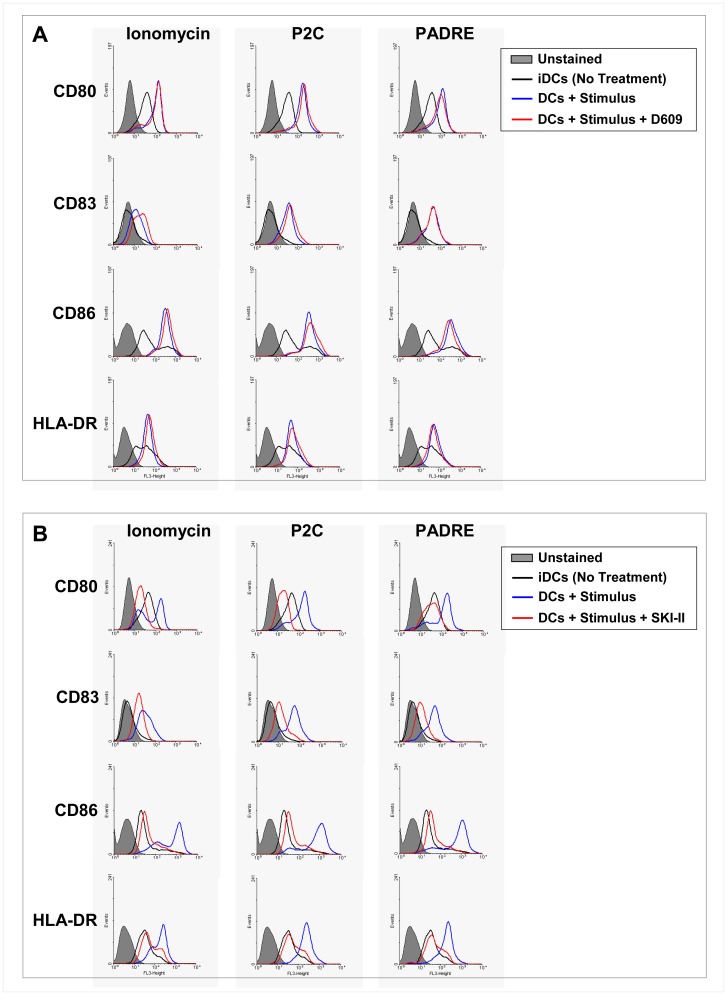
Maturation of DCs induced by particle-bound PADRE is via sphingosine kinase but not PLC. Effect of the PLC inhibitor D609 (**A**) and the sphingosine kinase inhibitor SKI-II (**B**) on the phenotypic maturation of DCs induced by particle-bound PADRE. Day-5 DCs were incubated with the inhibitors for 2 hours and then cultured with particle-bound PADRE, particle-bound P2C or ionomycin for 2 days and analyzed by flow cytometry for CD80, CD86, CD83 and HLA-DR. The histograms show the expression levels of a representative donor of at least three independent experiments done with cells from three different healthy donors.

Phospholipase C (PLC) cleaves phosphatidylinositol 4,5-bisphosphate into inositol triphosphate (IP3) and diacylglycerol (DAG). IP3 binds to its receptor on the endoplasmic reticulum triggering the release of calcium from these intracellular stores. PLC acts upstream of calcium release in signal transduction pathways. To determine whether signaling through PLC plays a role in the activation of DCs induced by particle-bound PADRE, we incubated day-5 iDCs with particle-bound PADRE, P2C and ionomycin with or without a prior 2 h incubation with the PLC inhibitor D609 at 100 µM. Two days later, the cells were harvested and analyzed by flow cytometry. There was no effect of the inhibitor on any of the maturation markers for DCs indicating that PLC has no role in DC activation by particle-bound PADRE (Figure 5A). Sphingosine 1-phosphate (S1P) has been shown to trigger the release of calcium from intracellular stores independent of IP3. S1P is derived from sphingosine through the phosphorylation of sphingosine by sphingosine kinase (SK). We blocked SK by incubating iDCs for 2 hours with the specific inhibitor SKI-II and then stimulated them with particle-bound PADRE, P2C or ionomycin. Interestingly, the cells treated with the SKI-II did not respond to any of the stimuli, pointing at a SK-dependent pathway of DC activation (Figure 5B).

### Maturation of DCs Induced by Particle-bound PADRE Involves Calmodulin but not Calcineurin

**Figure 6:**
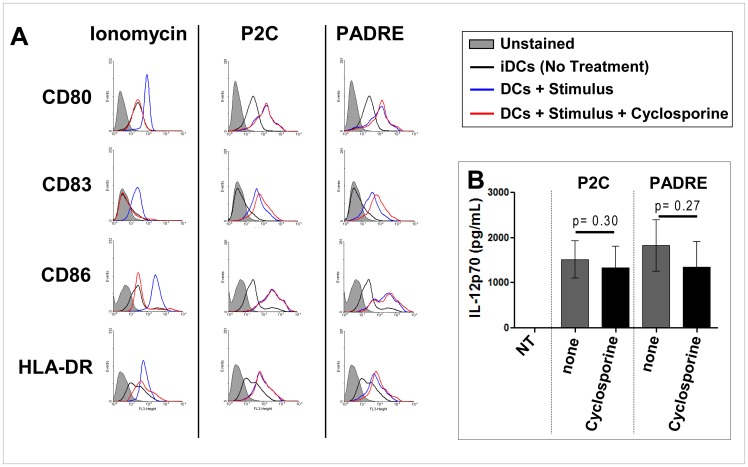
Maturation of DCs induced by particle-bound PADRE does not involve calcineurin. Day-5 DCs were collected and incubated with particle-bound PADRE, particle-bound P2C or ionomycin for additional 2 days in the presence of absence of cyclosporine A. On day 7 the cells were stained with antibodies for CD80, CD86, CD83 and HLA-DR, and analyzed by flow cytometry. The culture supernatants were analyzed for IL-12p70 by ELISA. The histograms in (**A**) show the expression of CD80, CD86, CD83 and HLA-DR of a representative donor, diagram (**B**) shows the secretion of IL-12p70. Independent experiments were done with cells from seven different healthy donors.

**Figure 7:**
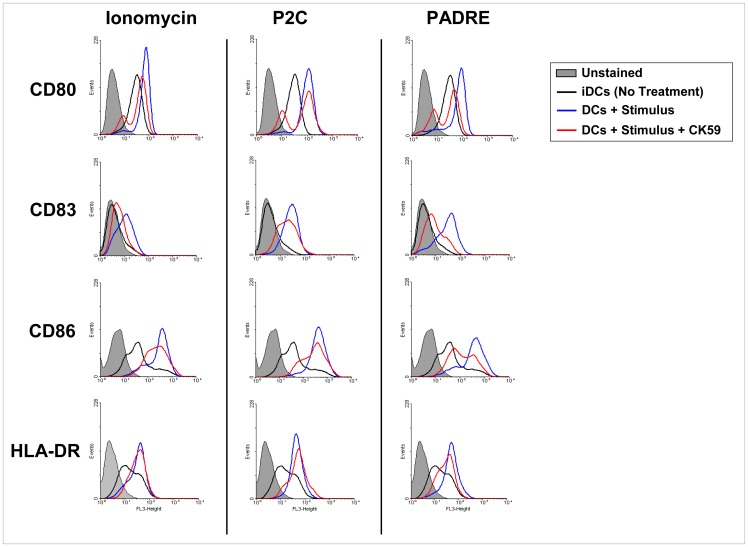
Maturation of DCs induced by particle-bound PADRE involves CaMKII. Effect of the CaMKII inhibitor CK59 on the phenotypic maturation of DCs induced by particle-bound PADRE. Day-5 DCs were cultured with particle-bound PADRE, particle-bound P2C or ionomycin for 2 days and analyzed by flow cytometry for CD80, CD86, CD83 and HLA-DR. The histograms show the expression levels of a representative donor of four independent experiments done with cells from four different healthy donors.

Given the essential role of cytosolic Ca^2+^ for DC activation by particle-bound PADRE, we investigated the involvement of the two mediators of Ca^2+^ signalling calcineurin and calmodulin. Inhibition of calcineurin with cyclosporin A had no effect on DCs activation by particle-bound PADRE (Figure 6); there was still up-regulation of CD83 and CD86 in the presence of cyclosporin A as it was the case in DCs stimulated with P2C-bearing particles but not in DCs incubated with the Ca^2+^ ionophore ionomycin. Also the secretion of IL-12p70 was unaltered. On the other hand, inhibition of the calmodulin pathway with CK59, an inhibitor of the calmodulin-dependent kinases CamKII, largely abrogated the expression of CD83 and substantially reduced the up-regulation of CD80, CD86 and HLA-DR by particle-bound PADRE (Figure 7). Partial inhibition of ionomycin-mediated induction of CD83 was seen; otherwise little to no effect was observed when DCs were incubated with ionomycin or particle-bound P2C in the presence of CK59, suggesting that different Ca^2+^-dependent pathways are involved in DC activation by the three stimulators tested. Thus, induction of DC maturation by particle-bound PADRE appears to involve calmodulin, whereas calcineurin seems to play a major role in the activation of DC by ionomycin. The TLR 2/6 agonist P2C acts through a different mechanism.

### Kinase Signaling in DC Activation by Particle-bound PADRE

**Figure 8:**
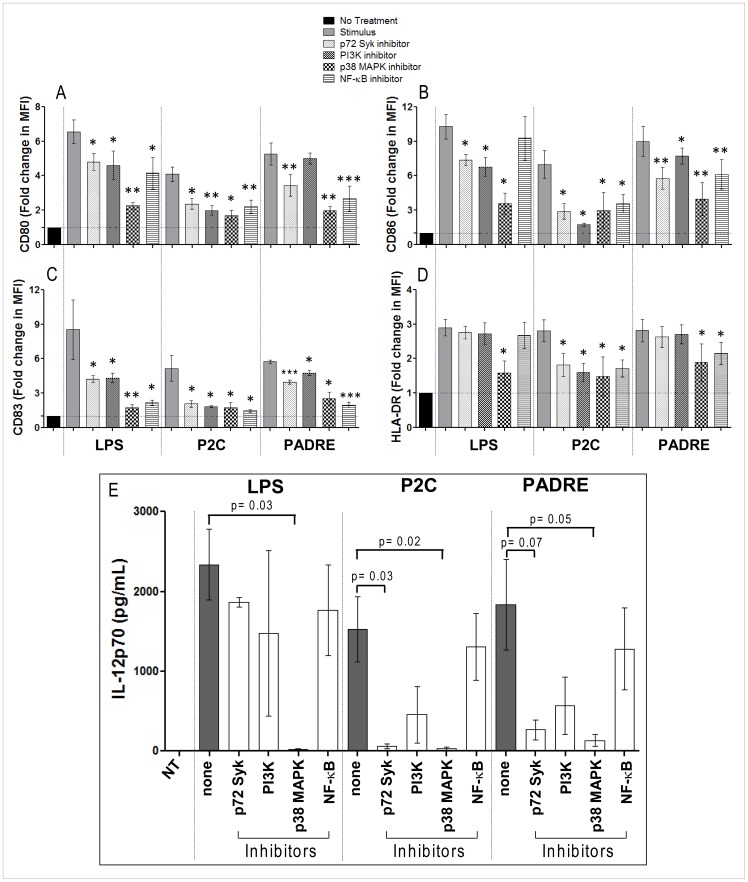
Effect of the specific inhibitors of kinase signaling on the phenotypic maturation of DCs induced by particle-bound PADRE. Day-5 DCs were incubated for 2 hours with inhibitors for p72 Syk, PI3K, p38 MAPK or NF-κB after which particle-bound PADRE, particle-bound P2C or soluble LPS were added and the cultures continued for 2 more days. On day 7 the cells were analyzed by flow cytometry for expression of CD80, CD86, CD83 and HLA-DR and their culture supernatant by ELISA for IL-12p70. The graphs show fold change of expression of CD80 (**A**), CD86 (**B**), CD83 (**C**) and HLA-DR (**D**), or IL-12p70 levels in the supernatants (**E**) with standard error of 7 independent experiments with cells from 7 different healthy donors. * = p<0.05; ** = p<0.01; and *** = p<0.001 comparing the group treated with stimulus only vs. the groups treated with stimulus plus the specific inhibitor.

The kinases p72Syk, PI3K and p38 MAPK have been described to play a significant role in DC maturation induced by different stimuli [22], [23] and the pathway they are involved in are interlinked with calcium signaling [24]–[26]. DC activation by particle-bound PADRE was affected by the inhibitors piceatannol and SB203580 that specifically block p72Syk and p38 MAPK, respectively, whereas the PI3K inhibitor LY294002 had little to no effect on the expression of the activation markers and co-stimulatory molecules, and the secretion of IL-12p70 by the DCs (Figure 8). The pattern of inhibition was similar to the effects of these factors on DC stimulation by the TLR 2/6 agonist P2C but different from LPS stimulation. Expectedly, blockade of the downstream transcription factor NF-κB with triptolide, an inhibitor of IkB degradation, had significant effects on the activation of the DC related to the activation markers and co-stimulatory molecules but not to the same extent on IL-12 secretion.

## Discussion

The results of the investigations described herein demonstrate that the MHC class II-binding pan-DR epitope PADRE, when administered in particle-bound form, acts directly on iDCs to induce their phenotypic and functional differentiation into mature DCs with potent T cell-stimulating capacity. Unlike DC activation by cognate antigen-dependent interaction with T cells, this effect was T cell-independent. The receptor for PADRE is HLA-DR [11 and 12], a MHC class II molecule. There is a growing body of evidence that MHC class II molecules are involved in signal transduction [23], [27]–[32]. Some antibodies with specificities for these molecules can activate MHC class II-expressing cells when cross-linking the molecules [27]. Also the MHC-II-ligand LAG-3, expressed on the surface of activated T cells was shown to induce DCs maturation [23]. The finding that DC activation by PADRE requires its coupling to particles could point into the same direction, that is, particle-bound PADRE may crosslink HLA-DR and thereby induces signaling into the cells with subsequent activation and maturation of immature into fully mature DCs. This signal is, as the experiments presented with this report show, similar in strength and effect on the DCs as signals induced by the known strong immune stimulator P2C, a TLR 2/6 agonist, or a cocktail of proinflammatory cytokines selected for their maximal effects on DCs.

Maturation of DCs involves a number of different signaling molecules and pathways [22], [33]. Triggering of DCs by various different ligands was shown to lead to a rise of intracellular calcium [33] pointing to a key role of calcium-modulated signaling pathways in the maturation process. Several different stimuli lead to increase of intracellular calcium in DCs [33], and calcium ionophores induce DCs maturation. Also exposure of DCs to particle-bound PADRE induced a raise in the intracellular calcium. This calcium was released from intracellular sources which, however, is not mediated through IP3 as in most other investigated situations, but through sphingosin phosphate. Sphingosin kinase, the enzyme that phosphorylates sphingosin, has been linked to signal transduction from MHC class II molecules. It is prominently expressed in DCs [34] which makes its involvement also in transduction of signals from other stimuli possible.

Calcium exerts its effects by binding to and modulating the function or activity of a variety of different proteins, some enzymes, some themselves modulators of the activity of other proteins [35]. Calcineurin [33], [35] and calmodulin [35], [36] are the most prominent mediators of calcium signaling. Calcineurin has been implicated in the activation of DCs, among others through activation of the transcription factor NFAT [35]. However, it played no role in the activation and maturation of DCs triggered by particle-bound PADRE. In contrast to calcineurin, calmodulin was shown to be involved in the activation of DCs by particle-bound PADRE, which is in agreement with recent reports of its involvement in DC maturation induced through other stimuli [37]. One of the target molecules of calcium-activated calmodulin is the calmodulin-dependent kinases II (CamKII). CamKII activation has been related to the activation of p38 MAPK [38] and NF-κB [39] which both have been shown to play important roles in DC maturation [22], [23]. In agreement with that, we show here that the inhibition of CamKII, p38 MAPK and NF-κB blocked the activation of DCs by particle-bound PADRE.

Based on the results of the experiments reported herein, we suggest the following model for the mechanism of DC activation by particle-bound PADRE. Particles with bound PADRE may crosslink HLA-DR at the surface of immature DCs, which triggers activation of the sphingosin kinase. The product of sphingosin kinase, phosphosphingosin, mediates calcium release from intracellular sources, which binds to and activates calmodulin, and via CamKII activates both the MAPK and the p72syk signaling pathways, which ultimately leads to activation of NF-κB and maybe other transcription factors and as result to activation of the DCs. The need to address both the p72syk and the MAPK signaling pathways for full activation and maturation of DCs has been suggested before and is confirmed with the present report.

The finding of DC activation through particle-bound particle reveals interesting new facets of MHC class II and its molecular cell biology and may have practical implications for the design of immune stimulators and vaccines. Particle-bound PADRE could be used as a new class of immunological adjuvant mediating T cell-dependent immune reactions where it may double as DC activator and helper T cell antigen. PADRE is often used in cancer immunotherapy protocols in the latter capacity to support induction of sustained CD8^+^ effector T cell responses, but so far only in free soluble form [13]–[16]. Binding to particles, as we show here may enhance its effect and lead to induction of additional DC functions known to be essential or at least highly beneficial for the induction of effective and sustained CD8^+^ effector T cell responses.

## Supporting Information

Figure S1 **Effects of T cells on the maturation of iDCs induced by particle-bound PADRE.** PBMCs separated by Ficoll gradient centrifugation were depleted of T cells or not, enriched for monocytes by adherence and cultured in serum-free AIM-V with IL-4 and GM-CSF. **A)** Percentage of CD4^+^ T cells present in one representative of four independent DC cultures. The panel on the left shows the dot plot from a DC culture where the T cells were not depleted, the right panel a DC culture where the T cells were depleted prior to culturing the monocytes for DC production. **B)** Phenotype of DCs from cultures previously depleted of T cells (white bars) or not (gray bars) prior to maturation with an inflammatory cytokine cocktail of IL-1β, IL-6 and TNF-α, particle-bound P2C or particle bound PADRE. The graphs show means with standard error of MFI for HLA-DR, CD40, CD80, CD83 and CD86, respectively, from four independent experiments with DCs prepared from PBMC of four different healthy donors.
(TIF)

Figure S2 **Proliferation of CD4^+^ T cells incubated with DCs matured with particle-bound PADRE, P2C or both, or an inflammatory cytokine cocktail of IL-1β, IL-6 and TNF-α.** Plastic-adherent PBMCs previously depleted of T cells were cultured in presence of GM-CSF and IL-4 for 5 days and then matured with the stimuli as indicated in the graph. The DCs obtained from the cultures were then cocultured with CFSE-labelled naïve CD4^+^ T cells for seven days. The cells were then stained for CD4, CD45RA and CD45RO, and analyzed by flow cytometry for CFSE levels as indicator for cell proliferation. The values shown refer to the percentage of CD4^+^CFSE^low^ cells and are the means of five independent experiments done with cells from five different healthy donors.
(TIF)
