# Supplementary figures and images for: Direct Activation of Human Dendritic Cells by Particle-Bound but Not Soluble MHC Class II Ligand
Source: PLoS One. 2013 May 2;8(5):e63039. doi: 10.1371/journal.pone.0063039 (PMC3642081; doi:10.1371/journal.pone.0063039)

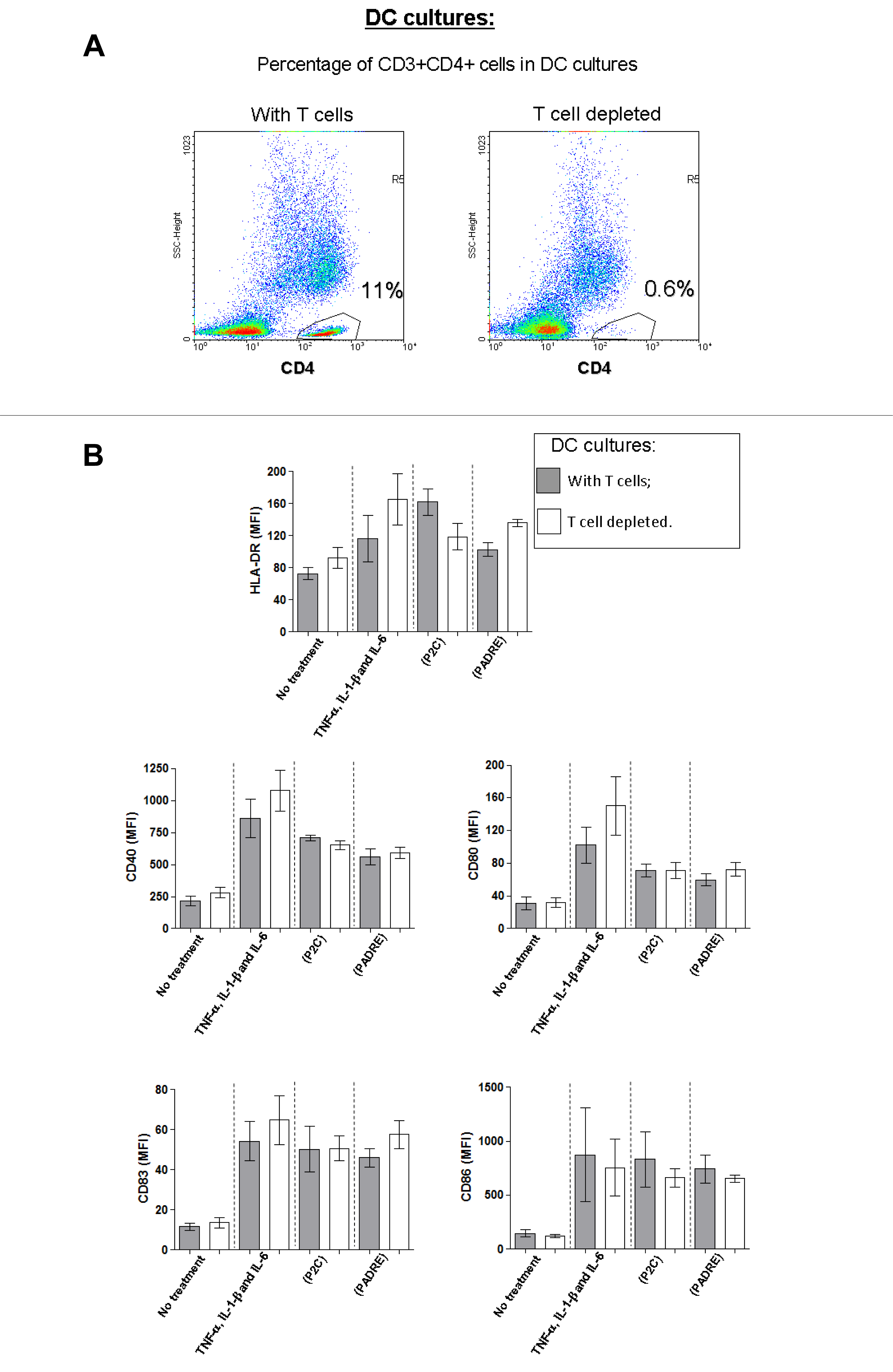

Supplement: Figure S1 — Effects of T cells on the maturation of iDCs induced by particle-bound PADRE. PBMCs separated by Ficoll gradient centrifugation were depleted of T cells or not, enriched for monocytes by adherence and cultured in serum-free AIM-V with IL-4 and GM-CSF. A) Percentage of CD4+ T cells present in one representative of four independent DC cultures. The panel on the left shows the dot plot from a DC culture where the T cells were not depleted, the right panel a DC culture where the T cells were depleted prior to culturing the monocytes for DC production. B) Phenotype of DCs from cultures previously depleted of T cells (white bars) or not (gray bars) prior to maturation with an inflammatory cytokine cocktail of IL-1β, IL-6 and TNF-α, particle-bound P2C or particle bound PADRE. The graphs show means with standard error of MFI for HLA-DR, CD40, CD80, CD83 and CD86, respectively, from four independent experiments with DCs prepared from PBMC of four different healthy donors. (TIF) [file pone.0063039.s001.tif]

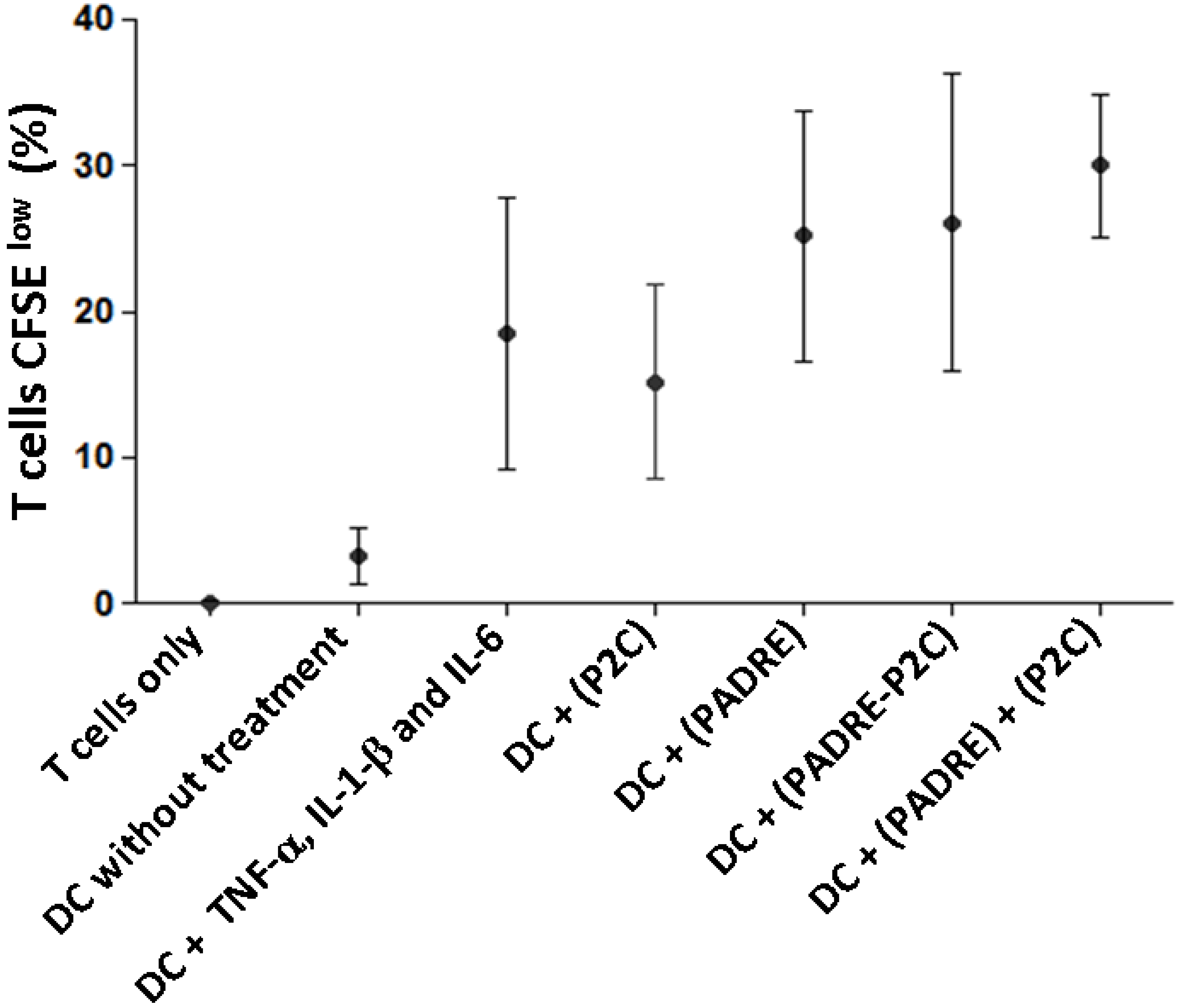

Supplement: Figure S2 — Proliferation of CD4+ T cells incubated with DCs matured with particle-bound PADRE, P2C or both, or an inflammatory cytokine cocktail of IL-1β, IL-6 and TNF-α. Plastic-adherent PBMCs previously depleted of T cells were cultured in presence of GM-CSF and IL-4 for 5 days and then matured with the stimuli as indicated in the graph. The DCs obtained from the cultures were then cocultured with CFSE-labelled naïve CD4+ T cells for seven days. The cells were then stained for CD4, CD45RA and CD45RO, and analyzed by flow cytometry for CFSE levels as indicator for cell proliferation. The values shown refer to the percentage of CD4+CFSElow cells and are the means of five independent experiments done with cells from five different healthy donors. (TIF) [file pone.0063039.s002.tif]
